# Pensions and Gratuities for Disabled Nurses

**Published:** 1919-10-25

**Authors:** 


### PENSIONS AND GRATUITIES FOR DISABLED NURSES.

### Correspondence between "The Hospital" and the Ministry of Pensions.

Copy] THE HOSPITAL,
28-29 Southampton Street, Strand, W.C. 2.
October 15, 1919.

SIR,—For some time past it has come to our knowledge that considerable uncertainty prevails among demobilised nursing sisters who have suffered disablement as to the proper course to pursue in order to obtain assistance from the military authorities. Under the Army Order dated August 1917 it would appear that a very efficient chain of relief, consisting of pension or gratuity, medical treatment, and technical training, has been arranged for their benefit. But many of these nurses are unaware of the

(Continued on page 86.)

**The Editor's Letter-Box**—*(continued from page 83).*

existence of this Order, and we are informed that when they apply for help to the Ministry of Pensions they find their application either altogether ignored or treated with slight attention after long delay. We shall be obliged if you will inform us to whom demobilised nurses ought to apply in order to obtain immediate investigation into their disabilities, and such pension, temporary grant, or fees for training in a new employment as they may prove to be entitled to. We shall be happy, in the interests of the nurses and in response to frequent inquiry, to give publicity through the medium of THE HOSPITAL to any information you can give us on the subject.

THE EDITOR.

To the Minister of Pensions.

